# Association between Vegetarian Diet Consumption and Academic Performance, Sleep Quality, and Health-Related Quality of Life among Female Undergraduate College Students

**DOI:** 10.1155/2024/5053639

**Published:** 2024-08-24

**Authors:** Rana F. Obeidat, Aisha S. Almadhaani, Amal K. Almemari, Ghareibah M. Alyammahi, Hamdah E. Alabdouli, Maryam M. Alhmoudi

**Affiliations:** Faculty of Health Sciences Higher Colleges of Technology, Fujairah, UAE

## Abstract

**Purpose:**

This cross-sectional survey study aimed to examine the association between vegetarian diet consumption and sleep quality, academic performance, and health-related quality of life among female undergraduate college students.

**Method:**

A sample of 158 undergraduate female college students was recruited using a convenience sampling approach. Data collection utilized reliable and validated English-language instruments including the Vegetarian Quality of Life Questionnaire (VEGQOL), Pittsburgh Sleep Quality Index (PSQI), and health-related quality of life scale (HRQoL). The data were analyzed using one-way analysis of variance (ANOVA) and appropriate descriptive statistics.

**Results:**

Vegetarians exhibited a significantly lower BMI compared to nonvegetarians (F (1, 156) = [6.09], *p*=0.015). Those strictly adhering to a vegan diet (48.79 ± 9.41) had the lowest vegetarian quality of life among all participants following various forms of a vegetarian diet (F (3, 68) = [2.78], *p*=0.04). The majority of female college students reported good to excellent general health (91.7%), with 53.2% perceiving their sleep quality as fairly good. However, the mean PSQI global score of 8.04 (±3.35) indicated poor sleep quality. No significant association was found between diet type (vegetarian vs. nonvegetarian) and academic performance (cGPA) (*χ*^2^ (4, *N* = 158) = 2.92, *p*=0.57). There was no significant relationship between diet types and academic performance, HRQoL, and sleep quality.

**Conclusion:**

Despite a significant association between vegetarian diet and lower BMI, surprisingly, no substantial relationships were identified between diet type and academic performance, HRQoL, and sleep quality. These findings contribute to the ongoing discourse on the potential impacts of a vegetarian diet on various facets of female college students' well-being and highlight the need for further exploration in this field.

## 1. Introduction

The term “vegetarian diet” encompasses all diets that do not include meat and fish, regardless of whether they incorporate other animal products such as dairy and eggs [1]. Vegetarian diets are abundant in nutrients primarily sourced from plant-based meals [2], and their global adoption is increasing due to health, environmental, religious, and ethical reasons [3]. This trend is also noticeable in the UAE, a culturally diverse country where vegetarian options are becoming more accepted due to exposure to different cultures [4].

Vegetarian diets have been linked to better physical and psychological well-being, reduced risk of diseases, and improved sleep quality [5–11]. For instance, certain foods like cherries, kiwis, and milk can promote sleep. These foods are notably abundant in tryptophan and B vitamins (i.e., B12), which enhance the production and secretion of the neuromodulator melatonin, regulating the biological clocks of awakening and sleeping cycles [8]. A vegetarian diet has the potential to positively impact health-related quality of life (HRQoL), encompassing physical, psychological, social, and environmental dimensions [11, 12]. Research indicates notable improvements in sleep quality, overall health status, and psychological well-being, including reduced anxiety and depression, among individuals adhering to a vegetarian diet [2, 13].

During the college phase, dietary choices can significantly influence academic performance, psychological well-being, and quality of life [14–16]. Vegetarian diets, known for their nutritional benefits, may contribute to better cognitive function, sleep patterns, and mental health among college students [16, 17]. Limited research has explored the collective influence of vegetarian diets on these variables, especially among female undergraduate college students in the UAE. Insights into the prevalence of vegetarian diet adoption and its potential connections with academic performance, sleep, and quality of life can offer valuable information for future educational and awareness campaigns aimed at encouraging healthier dietary habits among female undergraduate college students. Therefore, the primary aim of this study was to examine the associations between vegetarian diet consumption and sleep quality, academic performance, and HRQoL of life among female undergraduate college students. A secondary aim of the study was to examine the prevalence of vegetarian diet consumption among female undergraduate college students in the UAE.

## 2. Methods

### 2.1. Research Design

A cross-sectional survey design was used to examine associations between vegetarian diet and sleep quality, academic performance, and health-related quality of life among female undergraduate college students.

### 2.2. Sampling Procedure and Sample Size

Convenience sampling was utilized to recruit participants from the female undergraduate college student population at Higher Colleges of Technology, UAE. To examine the relationship between type of diet, academic performance, sleep quality, and quality of life among female undergraduate college students, one-way analysis of variance (ANOVA) was performed. Based on a priori sample size calculation, it was determined that a minimum sample size of 179 participants was necessary for the study. This calculation was based on a medium effect size of 0.25, a large sample power of 0.80, a two-tailed significance level (alpha) of 0.05, and four groups as the degree of freedom. Participants eligible for inclusion in this study were females aged 18 years or older, currently enrolled in college studies, and willing to participate. Newly enrolled students who had no cumulative grade point average (cGPA) were excluded from the study.

### 2.3. Data Collection Procedures

The collection of data commenced promptly upon obtaining approval from the HCT Research Ethics Integrity Committee (REIC). An electronic survey hosted on Microsoft Forms served as the means for data acquisition. HCT students were actively encouraged to participate through the dissemination of the survey link via announcements on the Blackboard learning platform, e-mail communications, and in-person interactions. All responses were directed to the principal investigators and securely stored on a password-protected device accessible exclusively to the study researchers. A thorough assessment was carried out to ensure completeness and accuracy, with careful attention given to addressing any missing information. The data collection process was terminated upon reaching the predefined minimum sample size. Data collection took place in October 2023.

### 2.4. Data Collection Tools

#### 2.4.1. Demographic Data

A questionnaire developed by researchers was used to collect general demographic data including age, gender, campus, semester, program, and cGPA.

#### 2.4.2. Vegetarian Diet

The Vegetarian Quality of Life Questionnaire (VEGQOL) [18] was employed to examine the prevalence of vegetarian diet consumption among participants and evaluate the quality of life among those adopting a vegetarian diet. The questionnaire, validated in English by the authors, exhibited satisfactory reliability (Cronbach's alpha of 0.70) and validity (content and discriminant validity). It comprised 19 questions in two sections—demographic variables (questions 1–6) and quality of life related to a vegetarian diet (questions 7–19). Response options for questions on vegetarian quality of life (i.e., questions 7–19) are on a five-point Likert scale ranging from 1 (i.e., never) to 5 (i.e., always). Quality of life scores are categorized as low (<60), regular (60–70), satisfactory (70–80), high (80–90), and very high (>90). The total score, ranging from 13 to 65, is obtained by summing all item scores, with reversed scoring for specific items (5, 7, 11, 12, 13, 14, and 15). The final score out of 100 is computed using the following equation:

(1) $$\begin{array}{c} \text{score}=\frac{\left(\text{sum of all item}s^{\prime }\text{score}\right)-13}{52}\;\times 100. \end{array}$$

#### 2.4.3. Health-Related Quality of Life

To assess health-related quality of life unrelated to a vegetarian diet, the health-related quality of life scale developed by the CDC [19] was employed. The questionnaire, validated in English, consists of 14 questions categorized into three sections: (1) basic health days; (2) physical, mental, and emotional problems; and (3) activities and health care. The scale utilizes diverse response formats, such as a 1 (excellent) to 5 (poor) scale for core healthy days and a yes or no format for the activity limitation module. The computation of the unhealthy days' score involves adding the number of physically unhealthy and mentally unhealthy days or subtracting the number of unhealthy days from 30, with a maximum score of 30 unhealthy days. The scale exhibits robust psychometric properties (i.e., content, construct, and criterion validity and reliability) and has been widely applied to evaluate quality of life across diverse ethnic and racial groups [20, 21].

#### 2.4.4. The Pittsburgh Sleep Quality Index (PSQI)

A widely utilized questionnaire, the Pittsburgh Sleep Quality Index (PSQI), assesses various facets of sleep through seven component scores and an overarching composite score [22]. These component scores encompass subjective sleep quality, sleep latency (the time it takes to fall asleep), sleep duration, habitual sleep efficiency (percentage of time asleep in bed), sleep disturbances, use of sleeping medication, and daytime dysfunction. Each item is assigned a weight on a 0–3 interval scale. The global PSQI score is derived by summing the seven component scores, resulting in an overall score ranging from 0 to 21, where lower scores indicate better sleep quality. PSQI demonstrated acceptable to good reliability, with Cronbach's alpha coefficients ranging from 0.64 to 0.83.

### 2.5. Data Analysis

All data were analyzed using the SPSS software (IBM SPSS Statistics, Version 29, 2023). Descriptive statistics were calculated in terms of counts and percentages for categorical variables and means and standard deviation (SD) for continuous variables. Several statistical tests were employed, including correlation tests and one-way analysis of variance (ANOVA). A *P* value of <0.05 was considered statistically significant in all analyses.

### 2.6. Ethical Considerations

The study was approved by the HCT Research Ethics and Integrity Committee (5-236RO). Participants were required to provide informed consent, confirming their willingness to participate in the study. This study adhered to the principles outlined in the Declaration of Helsinki.

## 3. Results

### 3.1. Demographic Characteristics of the Participants

A total of 180 individuals participated in and completed the online survey. However, 22 surveys were excluded due to significant missing data, resulting in a remaining set of 158 surveys suitable for analysis. Participants had a mean age of 20.3 years (±1.93). Most participants (92.4%) were affiliated with Fujairah Women's Campus and were enrolled in health science programs (61.4%). The largest group comprised fourth-year students (37.3%), followed by second-year students (30.4%). Approximately, 47% of participants had a cumulative grade point average (cGPA) between 2.1 and 3.

Among the respondents, 72 individuals adhered to a vegetarian diet, with an average BMI score of 21.24 (±3.70), whereas the 86 participants following a nonvegetarian diet had an average BMI of 22.90 (±4.60). Concerning the dietary patterns embraced by the participants, the majority (54.4%) identified as nonvegetarian, regularly consuming all types of meat. Among those who adopted a vegetarian diet, the majority, accounting for 20.2%, adhered to a standard vegetarian diet (i.e., not consuming any type of meat but consuming eggs and/or dairy products). Regarding the duration of adopting a vegetarian/vegan diet, 23 participants (31.9%) had consistently followed it, while 22 (30.6%) adopted it for less than 1 year. The primary motivation for adopting a vegetarian diet was personal health, as reported by 44 participants (61.1%). The One-Way Analysis of Variance (ANOVA) revealed significant differences in BMI between vegetarians and nonvegetarians. Vegetarians were more likely to have a lower BMI compared to nonvegetarians (F (1, 156) = [6.09], *p*=0.015). The demographic characteristics of the participants are presented in Table 1.

### 3.2. Vegetarian Quality of Life (VEGQOL) Descriptives

The VEGQOL scale was used to measure quality of life aspects directly associated with adhering to a vegetarian lifestyle, encompassing factors such as dietary satisfaction, social interactions, and personal beliefs related to vegetarianism. Participants who were following a vegetarian diet among the study participants (i.e., 72 participants) had a mean vegetarian quality of life (VEGQOL) score of 55.10 (±11.75), Table 2. The majority of those who were following a vegetarian diet had a low VEGQOL score, 51 (75%). The ANOVA test has shown that participants who were on a strict vegan diet (48.79 ± 9.41) had the lowest VEGQOL (F (3, 68) = [2.78], *p*=0.04) compared to others. There was no significant association between the time participants have been adopting a vegetarian diet (55.10 ± 11.75) and VEGQOL (F (3, 68) = [0.24], *p*=0.86).

### 3.3. Health-Related Quality of Life

The CDC health-related quality of life (QOL) scale was used to measure various dimensions of participants' well-being, including physical, mental, emotional, and social aspects, irrespective of their dietary choices. The majority of undergraduate female college students reported good general health (45.6%). However, there were a significant number of students who experienced between 3 and 5 unhealthy physical days over the past 30 days (21.5%). A total of 31 (19.7%) reported having a total of 3–5 unhealthy mental days over the past 30 days. A total of 61 (38.6%) of the participants had activity limitations for 3–9 days over the past 30 days. Moreover, during the past 30 days, participants had a mean score of 14.53 (±10.16) unhealthy days which include both physical health and mental health that kept them from doing usual activities such as self-care, work, or recreation, Table 3.

### 3.4. Pittsburgh Sleep Quality Index (PSQI)

Sleep characteristics, including the quality of sleep, the time it takes to fall asleep, the duration of sleep, sleep efficiency, and sleep disruption, were evaluated using PSQI. Participants in this study had a mean PSQI global score of 8.04 (±3.35), which indicates poor sleep quality.  Table 4 shows the PSQL results.

A total of 84 (53.2%) participants perceived their sleep quality as fairly good. Of all participants, only 15 (9.5%) reported having very bad quality of sleep. Regarding sleep latency, 80 (50.6%) participants indicated a sleep latency of less than 15 minutes, while 16 (10.1%) reported a sleep latency of more than 60 minutes. The majority of the participants (109, 69%) reported 85% or more sleep efficiency. A total of 56 (35.4%) of the participants reported a sleep duration of over 7 hours, while 21 participants (13.3%) reported having less than 5 hours of sleep duration. The majority of the participants (61%) went to bed after 10 pm and woke up between 5 and 7 am.

### 3.5. Relationship between Type of Diet, Academic Performance, Sleep Quality, and HRQoL

The results of the chi-square test revealed that there was no significant association between the type of diet (i.e., vegetarian vs. nonvegetarian) and academic performance (i.e., cGPA) (*χ*^2^ (4, *N* = 158) = 2.92, *p*=0.57). In addition, the ANOVA results indicated that there was no significant relationship between the type of diet and sleep quality (F (1,156) = [0.02], *p*=0.87) nor with any other aspects of sleep, such as sleep latency and sleep duration.

Similarly, the ANOVA results demonstrated that there was no significant association between the type of diet and HRQoL, as measured by unhealthy mental days (F (1, 155) = [0.13], *p*=0.71), unhealthy physical days (F (1, 156) = [0.08], *p*=0.77), and activity limitation (F (1, 156) = [1.14], *p*=0.28). Furthermore, the analysis showed that there was no significant relationship between the type of diet and the total number of unhealthy days (F (1, 156) = [0.35], *p*=0.55). These findings suggested that there was no significant association between type of diet (vegetarian vs. nonvegetarian), academic performance, sleep quality, and HRQoL.

## 4. Discussion

The study aimed to investigate the associations between vegetarian diet consumption, sleep quality, academic performance, and health-related quality of life (HRQoL) among female undergraduate college students. In addition, the study aimed to determine the prevalence of vegetarian diet consumption among this population in the UAE. The analysis of 158 suitable surveys showed that while 72 participants adhered to a vegetarian diet and had a lower average BMI, there was no significant association between vegetarian diet consumption and sleep quality, academic performance, or HRQoL.

Results of this study demonstrate a noteworthy correlation between dietary preferences (vegetarian vs. nonvegetarian) and BMI. Specifically, the results indicate that students who adhere to a vegetarian diet tend to have a lower BMI compared to those following a nonvegetarian dietary pattern. This observation aligns with the findings of Olfert et al. [23], whose study also reported that individuals practicing vegetarianism often exhibit lower BMI and smaller waist circumferences.

The reduced BMI observed among those adopting a vegetarian diet can be attributed to their deliberate decision to embrace a healthier dietary regimen, involving the avoidance of meat and high-fat foods, thereby yielding positive outcomes. A link between the consumption of fatty red meat and obesity, leading to higher BMI, has been consistently reported [24]. A lower BMI, serving as a favorable health indicator, has been associated with a decreased risk of cardiovascular disease and lower instances of obesity. Obesity, conversely, plays a role in the decline of physiological parameters, thus promoting the emergence and advancement of cardiovascular diseases like dyslipidemia and elevated blood glucose levels [25]. On the other hand, it is important to recognize that although BMI can provide a general measure of weight status, it may not accurately represent body composition, including lean muscle mass and the distribution of body fat [26]. A low BMI could be due to decreased lean mass resulting from dietary choices rather than overall healthy habits, which could skew the interpretation of our findings. Consequently, relying solely on BMI as a measure may not fully capture the complex relationship between diet and health outcomes. Thus, future research should consider incorporating more comprehensive measures of body composition, such as dual-energy X-ray absorptiometry (DXA), to better understand the impact of various diets on health and body composition.

Our study's findings indicate that participants who were adhering to a vegetarian diet had an overall low vegetarian quality of life (VEGQOL). Specifically, participants adhering to a strict vegan diet exhibited a significantly lower quality of life compared to those adopting other forms of vegetarian diets (e.g., pesco-vegetarian, semi-vegetarian). This finding contrasts with a study suggesting a positive correlation between vegan diet and improved health outcomes [27]. The diminished quality of life reported by vegetarians in this study, particularly strict vegans, may be due to their recent adoption of this dietary approach, leading to various adjustment challenges. Previous research [18] has found that vegetarians with longer adherence to the diet tend to report higher quality of life compared to those who have recently adopted it. Another potential reason for the lower quality of life among strict vegans could be the scarcity of suitable food options, particularly in the UAE and Middle Eastern regions, where traditional cuisine is heavily centered around rice and fatty meats. This dietary culture can create obstacles for those pursuing a vegan lifestyle, as compatible options may be limited. Furthermore, the high cost and restricted availability of vegetarian foods in certain areas could negatively affect quality of life, making it challenging to maintain a balanced and fulfilling diet [28].

The findings of this study indicate that there is no notable correlation between academic performance and the adherence to a vegetarian diet. This outcome diverges from the results of other studies [29, 30] which reported significant association between higher academic performance and better diet quality, including a greater consumption of fruits and vegetables. These conflicting findings may be attributed to various factors, including the potential for inaccurate self-reported data. In addition, it is noteworthy that a substantial portion of participants in this study were first-year students who may not have fully acclimated to college studies yet. Moreover, a significant number of participants were relatively new to adopting a vegetarian diet. Collectively, these factors may have contributed to the observed lack of a significant relationship between academic performance and vegetarian dietary patterns in this study.

In contrast to findings from other studies [10, 31], this research revealed no statistically significant association between sleep quality and the type of diet (vegetarian vs. nonvegetarian). Piekarska et al. [10] noted a positive correlation between fruit and vegetable consumption and the incidence of insomnia, suggesting that a higher intake of fruits and vegetables was linked to a lower rate of insomnia. This could potentially be attributed to individuals adhering to a vegetarian diet for an extended duration, as opposed to the participants in this study who, on average, had relatively short periods of following a vegetarian diet. While these findings hint at a potential relationship between diet and sleep quality, our study did not specifically observe such a correlation. One possible explanation for the absence of a significant association between diet type and sleep quality among the participants in this study could be the prevalent reporting of poor sleep quality, with a mean global Pittsburgh Sleep Quality Index (PASQ) score of 8.4.

Opting for a healthy diet reduces the risk of malnutrition and dietary deficiencies, and promotes normal bodily functions, contributing to an enhanced health-related quality of life (HRQoL), encompassing life satisfaction, physical well-being, and mental wellness [5, 6, 32]. Adopting a vegetarian diet can have positive effects on overall health and well-being. It has been consistently reported that a vegetarian diet, rich in fruits, vegetables, whole grains, and plant-based proteins, may lead to lower rates of chronic diseases such as heart disease, obesity, and certain types of cancer [6, 33]. Moreover, a vegetarian diet is often linked to higher intakes of fiber, vitamins, and minerals, supporting good health and enhancing the quality of life. In the context of this study, no significant relationship was observed between diet type and Quality of Life (QoL). These differing findings could potentially be attributed to factors such as the duration of adopting a vegetarian diet, or other elements contributing to a decrease in HRQoL among undergraduate college students, such as stress and/or study and life pressures [34]. Notably, the UAE generally boasts a high QoL, which aligns with this study's discovery that the majority of participants reported excellent to good general health (91.9%). Life satisfaction in the UAE was reported to be high among educated and affluent women, as well as among younger and older generations, married individuals, and those with successful career paths [35]. The country has made significant progress in various aspects of life, creating a positive and vibrant environment. While individual circumstances can influence QoL, overall, the UAE offers a favorable setting.

### 4.1. Strength and Limitations

It is essential to recognize both the strengths and limitations inherent in this study. A notable strength lies in the utilization of established and validated questionnaires with robust psychometric properties. However, several limitations should be acknowledged. First, the survey was presented in English, potentially impacting participants' comprehension, particularly since the majority were first-year students. Second, reliance on self-reported data introduces the possibility of bias and inaccuracies due to subjective perceptions, recall bias, and social desirability, potentially influencing result accuracy and internal validity. Third, another limitation of the study lies in its reliance solely on cumulative GPA (cGPA) as a measure of academic performance, potentially overlooking the broader spectrum of academic success. Factors such as class participation, engagement, and involvement in extracurricular activities could provide additional insights. Moreover, the analysis does not take into account external factors that impact academic performance and quality of life, such as study habits, stress levels, social support, and mental health. Fourth, the strictness of adherence to the type of diet and variability in dietary practices within each group were not evaluated. Fifth, the use of convenience sampling may introduce sampling bias, as those more accessible or with a greater online presence could be disproportionately represented. Finally, due to the cross-sectional design, no causal relationships can be inferred between diet type and the variables studied.

### 4.2. Recommendations

The study's findings offer several recommendations for consideration. First, it is important to enhance awareness among female college students regarding the adoption of a balanced diet that supports overall health and well-being. Encouraging students to maintain a diverse and well-balanced dietary regimen, regardless of whether it includes vegetarian or nonvegetarian options, can promote healthier lifestyle choices. This can be facilitated through the provision of educational tools, workshops, and access to dietitians. Incorporating educational courses at the beginning of the academic year, especially targeting first-year students, could effectively inform them about the significance of maintaining a healthy diet. Further, academic institutions play a pivotal role in facilitating students' adoption of a vegetarian diet, thereby enhancing their vegetarian as well as overall quality of life. To support this lifestyle choice, institutions can implement initiatives such as offering a diverse range of plant-based food options in campus dining facilities, establishing a supportive community, through vegetarian clubs or events which creates a sense of belonging for those adopting this lifestyle, and incorporating vegetarian-friendly options in campus events and activities to help normalize and accommodate diverse dietary preferences.

To address current research limitations, it is recommended that future studies adopt a more inclusive approach by recruiting a large, random sample of both male and female students from diverse higher academic institutions to enhance the generalizability of the findings. Utilizing an Arabic questionnaire for data collection is also suggested to enhance inclusivity. Future research should include objective measures (e.g., academic records for performance and clinical assessments for health), to ensure more reliable data. Future studies should include additional metrics to assess academic performance, such as class participation, engagement in coursework, and involvement in extracurricular activities. In addition, it is essential to consider and control for external factors influencing academic success and quality of life, such as study habits, stress levels, social support, and mental health. Employing more sophisticated statistical analyses, such as multivariate regression, can help achieve this. Accounting for these variables will provide a more comprehensive and accurate analysis. In future studies, it would be beneficial to focus on students who have committed to a healthy diet over an extended period. In addition, measuring the strictness of adherence to these diets and the variability in dietary practices within each group would provide a more comprehensive understanding. Lastly, a longitudinal study is crucial to explore potential causal relationships between a vegetarian diet, academic performance, sleep quality, and quality of life. Such research endeavors would contribute to expanding the existing knowledge base, providing further insights into both the potential advantages and challenges associated with vegetarianism.

## 5. Conclusion

In conclusion, this study explored the relationship between dietary patterns, academic performance, sleeping habits, and quality of life among female college students in the UAE. While the findings revealed no significant association between diet type and academic performance or sleep quality, the study shed light on the intricate interplay of factors influencing the quality of life. The limited duration of adopting a vegetarian diet, potential cultural and lifestyle challenges, and the prevalent poor sleep quality reported by participants underscore the need for further research. Recommendations include increasing awareness, providing educational resources, and conducting future studies with a broader sample and longitudinal design. By addressing these aspects, academic institutions can contribute to fostering a supportive environment for students embracing vegetarianism and promoting holistic well-being.

## Figures and Tables

**Table 1:** Demographic characteristics of the participants in the survey.

Variable	Category	Frequency	Percentage
Body mass index (BMI)	18.5 or less—underweight	30	19.0
	Between 18.6 and 24.9—normal	96	60.8
	Between 25.0 and 29.9—overweight	28	17.7
	30.0 or more—obese	4	2.5

Academic year	First year	36	22.8
	Second year	48	30.4
	Third year	15	9.5
	Fourth year	59	37.3

Program	Applied media	2	1.3
	Computer information science	20	12.7
	Education	2	1.3
	Engineering technology & science	10	6.3
	Health science	97	61.4
	Business	27	17.1

Campus	Fujairah women's campus	146	92.4
	Other campuses	12	7.6

cGPA	0.1–1	7	4.4
	1.1–2	11	7.0
	2.1–3	74	46.8
	3.1–4	66	41.8

Regarding the eating pattern you adopt, how do you classify it?	Vegan or strict vegetarian (not consuming any animal product)	16	10.1
	Vegetarian (not consuming any type of meat but consuming eggs and/or dairy products)	32	20.2
	Pesco-vegetarian (consuming fish/seafood but not consuming other types of meat)	8	5.0
	Semivegetarian (adopting a practically vegetarian diet but consuming meat less than once per week)	16	10.1
	Nonvegetarian (eating all types of meat on a frequent basis)	86	54.4

How long have you been adopting a vegetarian/vegan diet?	I have always adopted it	23	31.9
	For less than 1 year	22	30.6
	Between 1 and 5 years	17	23.6
	For more than 5 years	10	13.9

What was the MAIN motivation for you to adopt a vegetarian diet?	Ethical/moral reason (related to animals)	6	8.3
	Personal health	44	61.1
	Religion/beliefs/spirituality	7	9.7
	Environmental impact	10	13.9
	Influence of other people (family, friends, or close people)	4	5.6
	Other	1	1.4

**Table 2:** Frequency of VEGQOL score categories.

Category	Frequency	Percentage
Low	51	75.0
Regular	7	10.3
Satisfactory	7	10.3
High	2	2.9
Very high	1	1.5

**Table 3:** Health-related quality of life (QOL) descriptives.

Variable	Category	Frequency	Percent
Would you say that in general your health is?	Excellent	38	24.1
	Very good	35	22.2
	Good	72	45.6
	Fair	11	7.0
	Poor	2	1.3

Unhealthy physical days	0 days	21	13.3
	1–2 days	31	19.6
	3–5 days	34	21.5
	6–9 days	24	15.2
	10–19 days	33	20.9
	20–29 days	9	5.7
	All 30 days	6	3.8

Unhealthy mental days	0 days	20	12.7
	1–2 days	29	18.5
	3–5 days	31	19.7
	6–9 days	29	18.5
	10–19 days	19	12.1
	20–29 days	18	11.5
	All 30 days	11	7.0

Activity limitation	0 days	25	15.8
	1–2 days	25	15.8
	3–5 days	31	19.6
	6–9 days	30	19.0
	10–19 days	26	16.5
	20–29 days	15	9.5
	All 30 days	6	3.8
	0 days	25	15.8

**Table 4:** Pittsburgh Sleep Quality Index (PSQI) descriptives.

Statistics	*N* (%)
*Sleep quality*	
Very good	28 (17.7)
Fairly good	84 (53.2)
Fairly bad	31 (19.6)
Very bad	15 (9.5)

*Sleep latency*	
Less than 15 minutes	80 (50.6)
16–30 minutes	42 (26.6)
31–60 minutes	20 (12.7)
More than 60 minutes	16 (10.1)

*Sleep duration*	
More than 7 hours	56 (35.4)
6–7 hours	52 (32.9)
5–6 hours	29 (18.4)
Less than 5 hours	21 (13.3)

*Sleep efficiency*	
≥85%	109 (69.0)
75–84%	13 (8.2)
65–74%	10 (6.3)
<65%	26 (16.5)

*Sleep disturbance*	
Less than once a week	51 (32.3)
Once or twice a week	83 (52.5)
Three or more times a week	24 (15.2)

*Use of medication*	
Not during past month	99 (62.7)
Less than once a week	31 (19.6)
Once or twice a week	20 (12.7)
Three or more times a week	8 (5.1)

*Daytime dysfunction*	
Not a problem at all	36 (22.8)
Only a very slight problem	63 (39.9)
Somewhat a problem	52 (32.9)
A very big problem	7 (4.4)

## Data Availability

The data used to support the findings of this study are available from the corresponding author upon reasonable request.
